# Hypoxic pre-conditioning suppresses experimental autoimmune encephalomyelitis by modifying multiple properties of blood vessels

**DOI:** 10.1186/s40478-018-0590-5

**Published:** 2018-09-03

**Authors:** Sebok K. Halder, Ravi Kant, Richard Milner

**Affiliations:** Department of Molecular Medicine, MEM-151, The Scripps Research Institute, 10550 North Torrey Pines Road, La Jolla, CA 92037 USA

**Keywords:** Endothelial, Laminin, Hypoxic pre-conditioning, Experimental autoimmune encephalomyelitis, Blood-brain barrier

## Abstract

While hypoxic pre-conditioning protects against neurological disease the underlying mechanisms have yet to be fully defined. As chronic mild hypoxia (CMH, 10% O_2_) triggers profound vascular remodeling in the central nervous system (CNS), the goal of this study was to examine the protective potential of hypoxic pre-conditioning in the experimental autoimmune encephalomyelitis (EAE) model of multiple sclerosis (MS) and then determine how CMH influences vascular integrity and the underlying cellular and molecular mechanisms during EAE. We found that mice exposed to CMH at the same time as EAE induction were strongly protected against the development of EAE progression, as assessed both at the clinical level and at the histopathological level by reduced levels of inflammatory leukocyte infiltration, vascular breakdown and demyelination. Mechanistically, our studies indicate that CMH protects, at least in part, by enhancing several properties of blood vessels that contribute to vascular integrity, including reduced expression of the endothelial activation molecules VCAM-1 and ICAM-1, maintained expression of endothelial tight junction proteins ZO-1 and occludin, and upregulated expression of the leukocyte inhibitory protein laminin-111 in the vascular basement membrane. Taken together, these data suggest that optimization of BBB integrity is an important mechanism underlying the protective effect of hypoxic pre-conditioning.

## Introduction

Multiple sclerosis (MS) is the most common neurological disease in the young-middle age population, affecting more than 400,000 people in the United States [9, 44]. At the pathological level, MS is an autoimmune disease in which inflammatory leukocytes directed against myelin antigens, infiltrate the CNS (brain and spinal cord) to establish a chronic inflammatory response that results in demyelination and axonal degeneration [16, 27]. Strong evidence demonstrates that alterations in vascular properties at an early stage of disease onset play a central role in the initiation and maintenance of MS pathogenesis by permitting leukocyte infiltration into the CNS [18, 25, 39, 40]. CNS blood vessels are unique in forming an extremely tight barrier between the blood and parenchymal tissue (termed blood-brain barrier (BBB) or blood-spinal cord barrier (BSCB) depending on location), which confers high electrical resistance and low permeability properties, thus protecting neural cells from potentially harmful blood components [1, 23, 38]. The integrity of the BBB/BSCB is regulated at a number of levels including: (i) inter-endothelial tight junction proteins, (ii) the vascular basal lamina comprising a mixture of extracellular matrix (ECM) proteins, and (iii) the influence of astrocyte end-feet and pericytes [5, 7, 37, 45].

Pre-conditioning describes the phenomenon whereby exposure to a mild stimulus for a period of time confers protection against a subsequent stronger insult. In the study of ischemic stroke it is well established that pre-conditioning with mild hypoxia protects against the development of ischemic infarct [10, 34, 43]. Interestingly, several years ago, the Dore-Duffy lab demonstrated that hypoxic pre-conditioning may also protect against the progression of experimental autoimmune encephalomyelitis (EAE), an animal model of MS. [8] In that study, chronic exposure of mice to 10% O_2_ (chronic mild hypoxia, CMH) suppressed clinical severity of EAE, which correlated with reductions in both leukocyte adherence to cerebral blood vessels and their infiltration into the CNS. Despite the profound protection that hypoxic pre-conditioning affords, the cellular and molecular mechanisms underlying this protection have yet to be fully defined. Recent work suggests that CMH protects in part, by inducing an anti-inflammatory milieu in the spinal cord of EAE affected mice, as defined by reduced levels of infiltrating CD4+ T lymphocytes and delayed Th17-specific cytokine response, correlating with increased levels of regulatory T cells (Tregs) and the anti-inflammatory cytokine IL-10 [15].

In addition to exerting an anti-inflammatory effect on cells of the immune system, because CMH promotes a robust vascular remodeling response in the CNS [26, 30, 36], it is possible that it might also protect against EAE by modifying the properties of blood vessels. In particular, several lines of evidence suggest that CMH enhances the integrity of CNS blood vessels. First, CMH reduces the adherence of circulating leukocytes to the endothelium of cerebral blood vessels in mouse models of MS and ischemic stroke [8, 43]. Second, CMH triggers strong upregulation of endothelial tight junction proteins at the BBB [3, 20, 30]. Third, we have recently found that CMH also stimulates enhanced expression of the ECM protein laminin in the basement membranes of cerebral blood vessels [21], a protein which prevents leukocyte transmigration across blood vessel walls by virtue of its anti-adhesive properties on this cell type [41]. In light of these observations, the goal of this study was to examine the protective potential of CMH in a relapsing-remitting EAE model of MS and then determine how CMH influences vascular integrity and the different cellular and molecular components underlying this integrity. Specifically, we wanted to define how CMH influences vascular expression of the following parameters during EAE progression: (i) endothelial activation molecules that mediate leukocyte adherence to the endothelium, vascular cell adhesion molecule-1 (VCAM-1) and intercellular adhesion molecule-1 (ICAM-1), (ii) the tight junction proteins ZO-1 and occludin, and (iii) vascular ECM proteins thought to be important for regulating leukocyte infiltration into the CNS.

## Materials and methods

### Animals

The studies described have been reviewed and approved by The Scripps Research Institute (TSRI) Institutional Animal Care and Use Committee. Wild-type female SJL/J mice were purchased from JAX labs and maintained under pathogen-free conditions in the closed breeding colony of TSRI.

### Experimental autoimmune encephalomyelitis (EAE)

EAE was performed using a protocol and materials provided by Hooke Laboratories (Lawrence, MA). Briefly, 10 week old SJL/J female mice were immunized sub-cutaneously with 200 μl of 1 mg/ml PLP_139–151_ peptide emulsified in complete Freund’s adjuvant (CFA) containing 1 mg/ml Mycobacterium tuberculosis in both the base of the tail and upper back. This protocol leads to robust induction of clinical EAE 10–15 days following immunization in which mice reach peak disease before making significant recovery (remission), but then follow a cyclical relapsing-remitting course [32, 33]. Animals were monitored daily for clinical signs and scored as follows: 0-no symptoms; 1-flaccid tail; 2-paresis of hind limbs; 3-paralysis of hind limbs; 4-quadriplegia; 5-death. Clinical EAE data were assessed using one-way analysis of variance (ANOVA) followed by post-hoc Student’s test, in which *p* < 0.05 was defined as statistically significant. For histological analysis, disease-free controls or EAE mice maintained under normoxic or hypoxic conditions were euthanized during the peak phase of disease, typically 14–15 days post-immunization.

### Chronic hypoxia model

Following immunization with PLP_139–151_ peptide, mice, housed 4 to a cage, were randomly divided into two groups: one was placed into a hypoxic chamber (Biospherix, Redfield, NY) maintained at 10% O_2_ for the duration of the experiment, while the control group was kept in the same room under similar conditions except that they were kept at ambient sea-level oxygen levels (normoxia, approximately 21% O_2_ at sea-level). Every day, the chamber was briefly opened to allow for clinical assessment of mice and cage cleaning and food and water replacement.

### Immunohistochemistry and antibodies

Immunohistochemistry was performed on 10 μm frozen sections of cold phosphate buffer saline (PBS) perfused tissues as described previously [35]. Rat monoclonal antibodies from BD Pharmingen (La Jolla, CA) reactive for the following antigens were used in this study: CD31 (clone MEC13.3), MECA-32, VCAM-1 (clone 429), and CD45. Rat monoclonal reactive for CD4 (clone GK1.5) was obtained from R&D Systems, Minneapolis, MN). Hamster monoclonal antibodies used included CD31 (clone 2H8) from Abcam (Cambridge, MA) and ICAM-1 (clone 3E2) from BD Pharmingen. The mouse monoclonal antibody (4H-8) against the anti-laminin α2 subunit was obtained from Sigma (St. Louis, MO). Rabbit polyclonal antibodies reactive for the following proteins were also used: occludin and ZO-1 (all from Invitrogen, Carlsbad, CA), fibrinogen (Millipore, Temecula, CA), and pan-laminin (Sigma, St. Louis, MO). In addition, the rabbit polyclonal against the anti-laminin α1 subunit was a kind gift from Dr. Takako Sasaki (Oita University, Japan). The goat antibody reactive for collagen IV was obtained from Millipore. Fluoromyelin-red was obtained from Invitrogen. Secondary antibodies used included Cy3-conjugated anti-rat, anti-rabbit, anti-goat and anti-mouse and Cy5-conjugated anti-rabbit from Jackson Immunoresearch, (West Grove, PA) and Alexa Fluor 488-conjugated anti-rat, anti-hamster and anti-rabbit from Invitrogen (Carlsbad, CA).

### Image analysis

Images were taken using a 2×, 10× or 20× objective on a Keyence 710 fluorescent microscope. All analysis was performed in the lumbar spinal cord. For each antigen, images of three randomly selected areas were taken at 10× or 20× magnification and three sections per spinal cord analyzed to calculate the mean for each subject. For each antigen in each experiment, exposure time was set to convey the maximum amount of information without saturating the image. Exposure time was maintained constant for each antigen across the different experimental groups. To quantify the degree of CD45+ or CD4+ leukocyte infiltration, fibrinogen leakage, vascular expression of ICAM-1 and VCAM-1, the tight junction proteins ZO-1 and occludin, and expression of laminins and collagen IV within blood vessels, NIH Image J software was used to measure the total fluorescent signal per field of view (FOV). Each experiment was performed with 6 different animals per condition, and the results expressed as the mean ± SEM. Statistical significance was assessed using one-way analysis of variance (ANOVA) followed by Tukey’s multiple comparison post-hoc test, in which *p* < 0.05 was defined as statistically significant.

## Results

### Hypoxic pre-conditioning reduces the severity of EAE both clinically and histopathologically

**Fig. 1:**
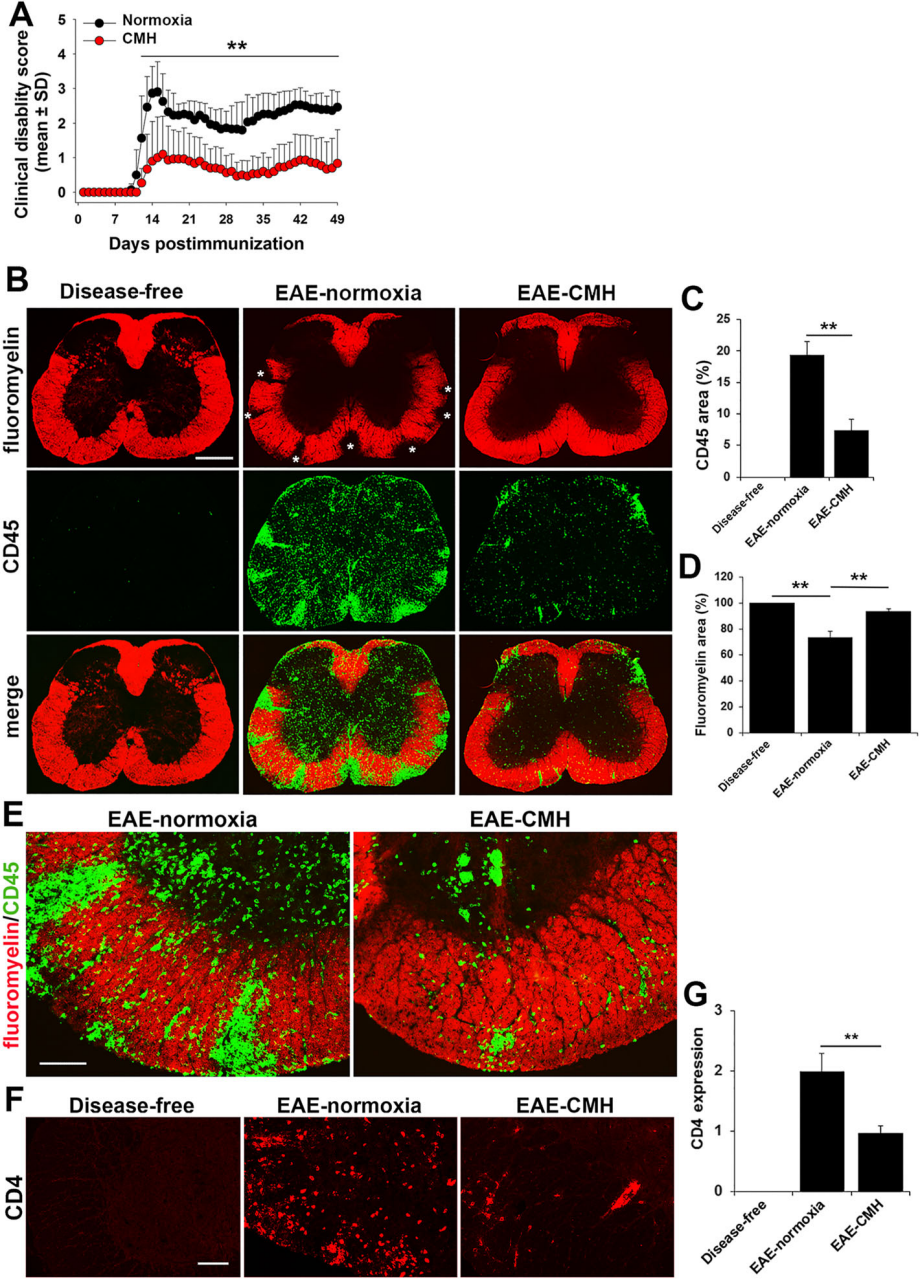
Hypoxic pre-conditioning reduces the severity of EAE both clinically and histopathologically. **a**. The impact of chronic mild hypoxia (CMH) on clinical severity in EAE. The progression of EAE in mice maintained under normoxic (control) or CMH conditions was evaluated by measuring clinical score at daily intervals. All points represent the mean ± SD (*n* = 15 mice per group, representative of 4 separate experiments). Note that compared to normoxic mice, CMH markedly reduced clinical score both at the peak of disease activity and at all time-points thereafter for the duration of the experiment (7 weeks), resulting in a marked and sustained reduction in long-term clinical score. **b**, **e** and **f**. Frozen sections of lumbar spinal cord taken from disease-free, EAE-normoxia or EAE-CMH mice at the peak symptomatic phase of EAE (14–15 days post-immunization) were stained for the inflammatory leukocyte marker CD45 (AlexaFluor-488) and fluoromyelin-red (FM) in panels B (scale bar = 500 μm) and E (scale bar = 100 μm) or CD4 in panel F (scale bar = 100 μm). Quantification of CD45 (**c**), fluoromyelin (**d**) and CD4 (**g**) fluorescent signal at peak phase of EAE. Results are expressed as the mean ± SEM (*n* = 6 mice/group). Note that CMH markedly suppressed CD45+ and CD4+ leukocyte infiltration and protected against demyelination. In panel B asterisks mark the zones of demyelination. ** *p* < 0.01

Previous studies have shown that hypoxic pre-conditioning delays the time of onset of the chronic progressive form of EAE [8, 15]. In the current study we chose to examine the influence of hypoxic pre-conditioning in the relapsing-remitting form of EAE for two reasons. First, the relapsing-remitting form of MS constitutes more than 85% of patients, so this model has strong translational relevance [4]. Second, in contrast to the chronic progressive model, in which mice reach peak disease and then only partially recover, in the relapsing-remitting model, mice reach peak disease before making significant recovery, but then follow a cyclical relapsing-remitting course [32, 33]. Ten weeks old female SJL/J mice were immunized with PLP_139–151_ and maintained under normoxic conditions or exposed to chronic mild hypoxia (CMH) at 10% O_2_ for the duration of the experiment. As shown in Fig. 1a, CMH markedly reduced clinical score both at the peak of disease activity and at all time-points thereafter for the duration of the experiment (7 weeks), resulting in a marked and sustained reduction in long-term clinical score. Histopathological assessment of spinal cord tissue with the pan-leukocyte marker CD45 and the myelin stain fluoromyelin (CD45/fluoromyelin dual-IF) revealed that compared to normoxic EAE mice, CMH-treated EAE mice showed marked reduction in the level of CD45+ leukocyte infiltration into the spinal cord (Fig. 1b). Quantification revealed that CMH significantly reduced CD45+ leukocyte infiltration (7.37 ± 1.72 vs. 19.40 ± 3.06% total CD45+ area/FOV under normoxic conditions, *p* < 0.01) (Fig. 1c) and this was associated with preservation of myelin (93.67 ± 2.11 vs. 73.74 ± 4.15% of fluoromyelin area/FOV under normoxic conditions, *p* < 0.01) (Fig. 1d). In addition, CD4 IF staining revealed that while CD4+ T cells were widely distributed in the spinal cord of EAE-normoxic mice, in EAE-CMH mice, they were tightly clustered and largely contained within perivascular aggregates (Fig. 1f-g). Compared to normoxic conditions, CMH markedly reduced CD4+ leukocyte infiltration into the spinal cord (0.96 ± 0.12 fluorescent units/FOV vs. 1.99 ± 0.29, *p* < 0.01). This demonstrates that CMH markedly suppressed EAE progression, both at the clinical and histopathological levels.

**Fig. 2:**
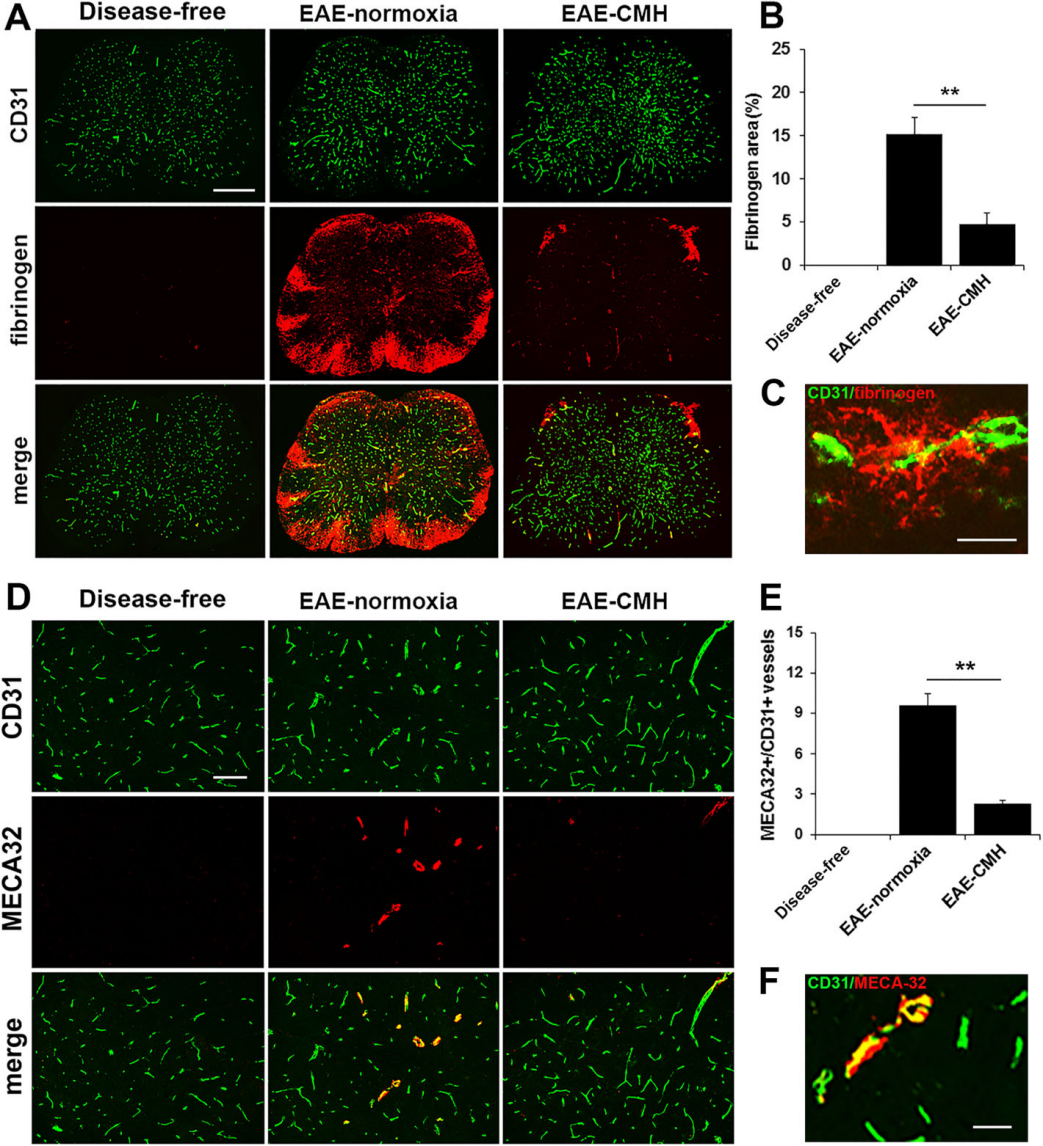
CMH protects against loss of vascular integrity during EAE progression. **a** and **d**. Frozen sections of lumbar spinal cord taken from disease-free, EAE-normoxia or EAE-CMH mice at the peak symptomatic phase of EAE were stained for CD31 (AlexaFluor-488) and fibrinogen (Fbg) (Cy-3) in panel A (Scale bar = 500 μm) or CD31 (AlexaFluor-488) and MECA-32 (Cy-3) in panel D (Scale bar = 100 μm). **b** and **e**. Quantification of fibrinogen leakage (**b**) and MECA-32 expression (**e**). Results are expressed as the mean ± SEM (*n* = 6 mice/group). **c** and **f**. High power images of CD31/fibrinogen (**c**) and CD31/MECA-32 (**f**). Scale bar = 25 μm. Note that CMH markedly suppressed fibrinogen leakage as well as expression of MECA-32. ** *p* < 0.01

Interestingly, we noticed that the distribution of CD45+ leukocytes within the spinal cords of normoxic and CMH-treated mice differed in two respects. First, under normoxic conditions, the greatest accumulation of leukocytes was in the ventral spinal cord, while under CMH conditions, most were found in the dorsal region (Fig. 1b). Second, closer inspection of high power images (Fig. 1e) revealed that while in the normoxic spinal cord, inflammatory CD45+ leukocytes were dispersed throughout the spinal cord white and gray matter, in CMH-treated mice they were mostly organized in large perivascular aggregates and leukocyte dispersal was limited. Based on this observation, we wondered if CMH might be protecting against EAE by restricting the ability of leukocytes to cross the blood-spinal cord barrier (BSCB). To examine this process more deeply, specifically to define how CMH influences vascular integrity, we, performed dual-IF with the endothelial cell marker CD31 and fibrinogen, using fibrinogen leakage as a marker of vascular breakdown (Fig. 2a-c). This revealed that while spinal cords of EAE-normoxic mice had extensive fibrinogen leakage, most notably in white matter, and strongly localized with inflammatory infiltrates, tissue from EAE-CMH mice showed significantly reduced levels of fibrinogen leak (4.71 ± 1.32 compared to 15.17 ± 1.95% total fibrinogen area/FOV under normoxic conditions, *p* < 0.01) (Fig. 2b). In an alternative approach to examine whether blood vessels in CMH-treated mice have altered barrier properties, we also analyzed expression of MECA-32, a marker that is expressed at high levels on endothelial cells in the developing CNS, but then disappears as CNS endothelium matures [22]. Previous studies have shown that MECA-32 is re-expressed in adult CNS blood vessels during inflammatory, hypoxic or ischemic conditions [11, 29, 42], suggesting that MECA-32 can be used to identify CNS blood vessels with compromised vascular integrity. Our analysis showed that while no MECA-32 staining could be detected in disease-free spinal cord, significant vascular staining for MECA-32 was detected in EAE mice maintained under normoxic conditions (Fig. 2d-f). Importantly, blood vessels in EAE mice maintained under CMH conditions showed significantly less MECA-32 signal than those maintained under normoxic conditions (2.28 ± 0.23 compared to 9.61 ± 0.87 MECA-32+ vessels/FOV, *p* < 0.01). Taken together, these findings demonstrate that CNS blood vessels in CMH-treated mice show less vascular breakdown, thus suppressing leukocyte infiltration and the progression of EAE.

### CMH suppresses endothelial expression of VCAM-1 and ICAM-1 during EAE

**Fig. 3:**
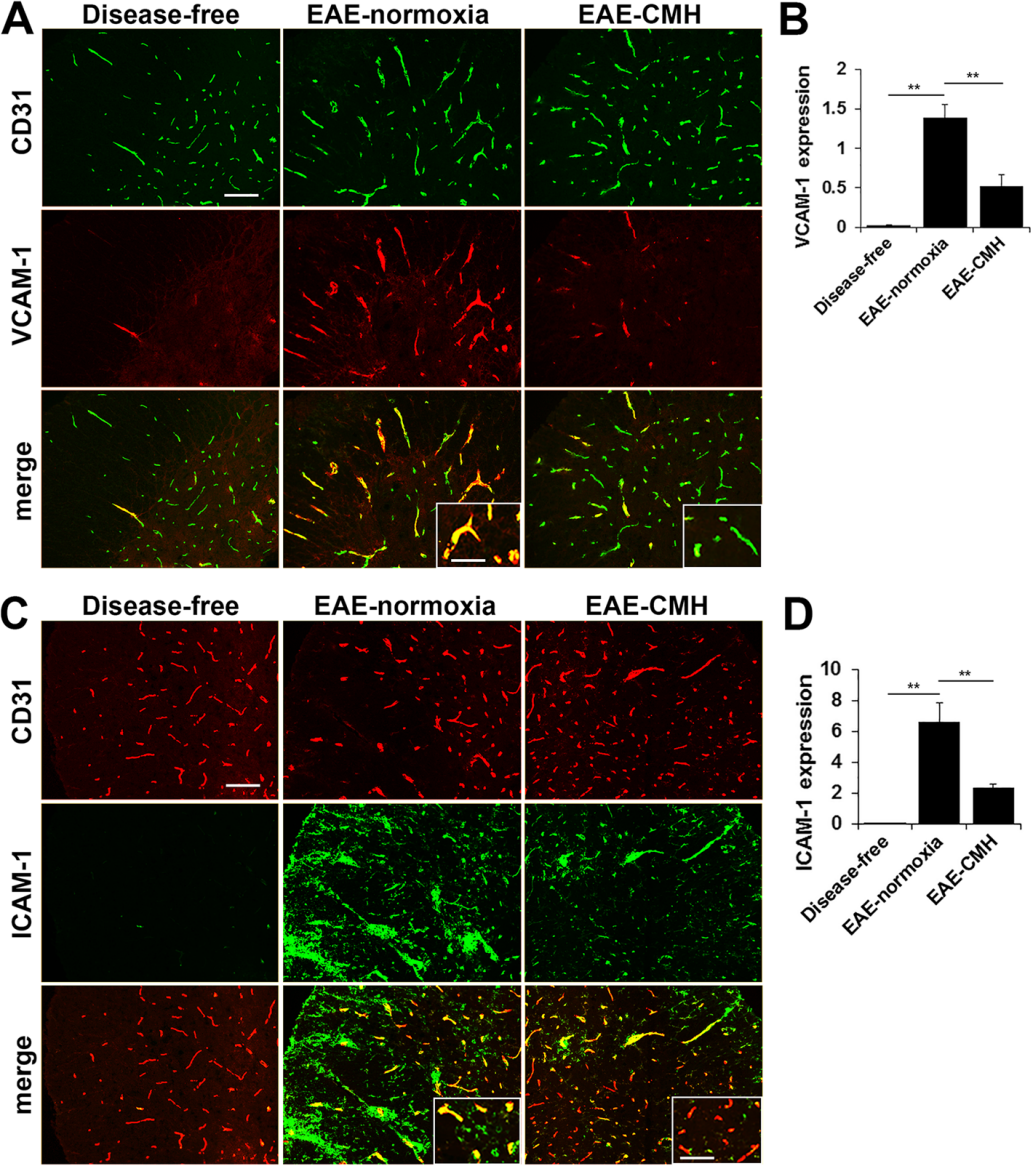
CMH suppresses endothelial VCAM-1 and ICAM-1 expression during EAE. **a** and **c**. Frozen sections of lumbar spinal cord taken from disease-free, EAE-normoxia or EAE-CMH mice at the peak symptomatic phase of EAE were stained for CD31 (AlexaFluor-488) and VCAM-1 (Cy-3) in panel A or CD31 (Cy-3) and ICAM-1 (AlexaFluor-488) in panel C. Scale bar = 100 μm (inset, scale bar = 50 μm). **b** and **d**. Quantification of VCAM-1 (**b**) and ICAM-1 expression (**d**). Results are expressed as the mean ± SEM (*n* = 6 mice/group). Note that CMH markedly suppressed vascular expression of VCAM-1 and ICAM-1. ** *p* < 0.01

An important clue suggesting how hypoxic pre-conditioning might be attenuating neuroinflammation was presented by the finding that CMH reduces the adhesion of circulating leukocytes to the endothelium of cerebral blood vessels in mouse models of MS and ischemic stroke [8, 43]. To transmigrate across the blood vessel wall, infiltrating leukocytes use integrin adhesion molecules (predominantly α4β1 and αLβ2 and αMβ2 integrins) to bind to counter-receptors (vascular cell adhesion molecule-1 (VCAM-1) and intercellular adhesion molecule-1 (ICAM-1)) expressed on the luminal side of endothelial cells lining blood vessels [28]. Under normal resting conditions, endothelial expression of VCAM-1 and ICAM-1 are barely detectable, but following endothelial activation, their expression is strongly induced [28]. To examine whether CMH might be inhibiting leukocyte adhesion to blood vessel walls by suppressing endothelial VCAM-1 and ICAM-1 expression, we performed dual-IF with VCAM-1/CD31 and ICAM-1/CD31 on spinal cord tissue obtained from mice that were either disease-free or had EAE, maintained under normoxic or CMH conditions. This revealed that VCAM-1 expression was strongly upregulated on spinal cord blood vessels in EAE mice maintained under normoxic conditions (from 0.03 ± 0.02 fluorescent units/FOV under disease-free conditions to 1.38 ± 0.17 at the peak stage of EAE under normoxic conditions, *p* < 0.01), but these levels were markedly suppressed in CMH-treated mice (0.53 ± 0.13 fluorescent units/FOV vs. 1.38 ± 0.17, *p* < 0.01) (Fig. 3a-b). ICAM-1 is expressed not only by activated endothelial cells but also by inflammatory leukocytes, making interpretation more difficult. However, Fig. 3c clearly shows that while ICAM-1 is absent in the spinal cords of disease-free mice, EAE-normoxic mice show strong upregulation of ICAM-1 both on infiltrating leukocytes and on activated blood vessels. Importantly, by examining areas of the spinal cord lacking leukocyte infiltration (see insets in Fig. 3c), we observed that ICAM-1 was strongly upregulated on spinal cord blood vessels in normoxic-EAE mice (from 0.03 ± 0.01 fluorescent units/FOV under disease-free conditions to 6.62 ± 1.21 at the peak stage of EAE under normoxic conditions, *p* < 0.01), but this expression was markedly suppressed in CMH-treated EAE mice (2.33 ± 0.26 fluorescent units/FOV vs. 6.62 ± 1.21, *p* < 0.01) (Fig. 3c-d). Thus in the EAE model, CMH suppresses endothelial expression of the activation molecules VCAM-1 and ICAM-1.

### CMH protects against loss of the endothelial tight junction proteins ZO-1 and occludin during EAE

**Fig. 4:**
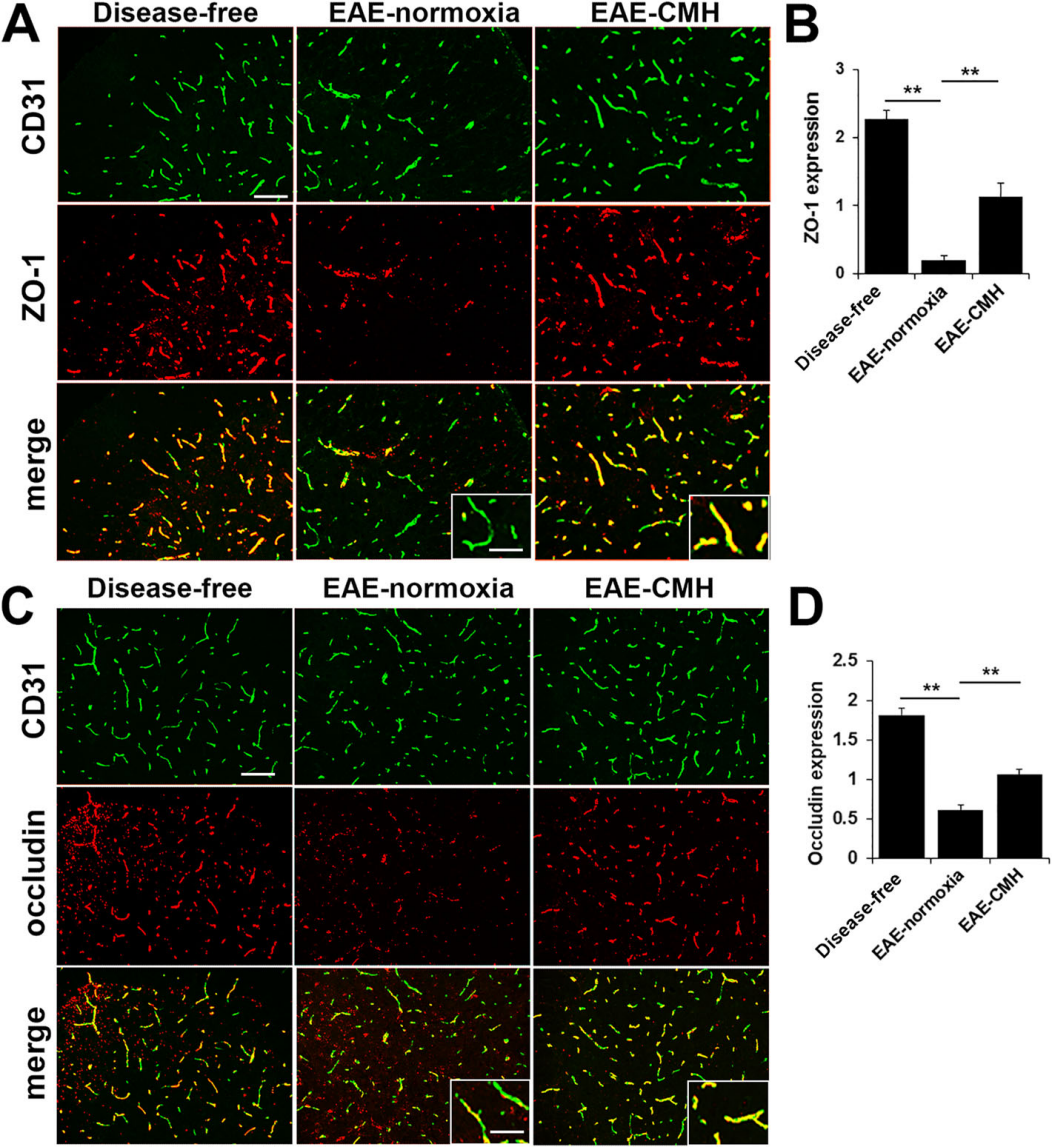
CMH protects against loss of the endothelial tight junction proteins ZO-1 and occludin during EAE. **a** and **c**. Frozen sections of lumbar spinal cord taken from disease-free, EAE-normoxia or EAE-CMH mice at the peak symptomatic phase of EAE were stained for CD31 (AlexaFluor-488) and ZO-1 (Cy-3) in panel A or CD31 (AlexaFluor-488) and occludin (Cy-3) in panel C. Scale bar = 100 μm (inset, scale bar = 50 μm). **b** and **d**. Quantification of ZO-1 (**b**) and occludin expression (**d**). Results are expressed as the mean ± SEM (*n* = 6 mice/group). Note that CMH protected against loss of ZO-1 and occludin. ** *p* < 0.01

The vascular integrity of CNS blood vessels is highly dependent on endothelial expression of tight junction proteins such as ZO-1 and occludin, which form tight connections between neighboring endothelial cells [1, 23, 45]. Two reasons suggest this may be relevant to CMH-mediated protection against EAE. First, expression of tight junction proteins at endothelial cell-cell borders is known to be disrupted both in MS and in EAE [2, 14, 25]. Second, we previously demonstrated that CMH triggers strong upregulation of tight junction proteins on CNS blood vessels, both in the brain and spinal cord [3, 20, 30]. Therefore, to examine how CMH treatment influences endothelial expression of tight junction proteins in the EAE model, we performed dual-IF of CD31/ZO-1 or CD31/occludin on spinal cord sections taken from CMH or normoxic mice at the peak stage of EAE. As expected, under disease-free conditions, ZO-1 and occludin co-localized tightly with the endothelial cell marker CD31 on all blood vessels (Fig. 4). However, during the peak stage of EAE in normoxic mice, vascular expression levels of ZO-1 and occludin were significantly reduced compared to disease-free control levels (Fig. 4). In contrast, blood vessels in CMH-treated EAE mice retained significant expression of ZO-1 and occludin. Quantification revealed that vascular ZO-1 levels were reduced from 2.28 ± 0.12 fluorescent units/FOV under disease-free conditions to 0.19 ± 0.07 at the peak stage of EAE under normoxic conditions (*p* < 0.01) but at the same time-point, ZO-1 levels in CMH-treated EAE mice were significantly higher than normoxic EAE mice (1.14 ± 0.2 fluorescent units/FOV vs. 0.19 ± 0.07, *p* < 0.01). In a similar manner, vascular occludin levels were reduced from 1.81 ± 0.08 fluorescent units/FOV under disease-free conditions to 0.61 ± 0.06 at the peak stage of EAE under normoxic conditions (*p* < 0.01) but at the same time-point, occludin levels in CMH-treated EAE mice were significantly higher than normoxic EAE mice (1.05 ± 0.07 fluorescent units/FOV vs. 0.61 ± 0.06, *p* < 0.01). These data demonstrate that CMH protects against loss of the endothelial tight junction proteins ZO-1 and occludin during EAE pathogenesis.

### CMH enhances laminin-111 expression in the parenchymal vascular basement membrane in EAE

**Fig. 5:**
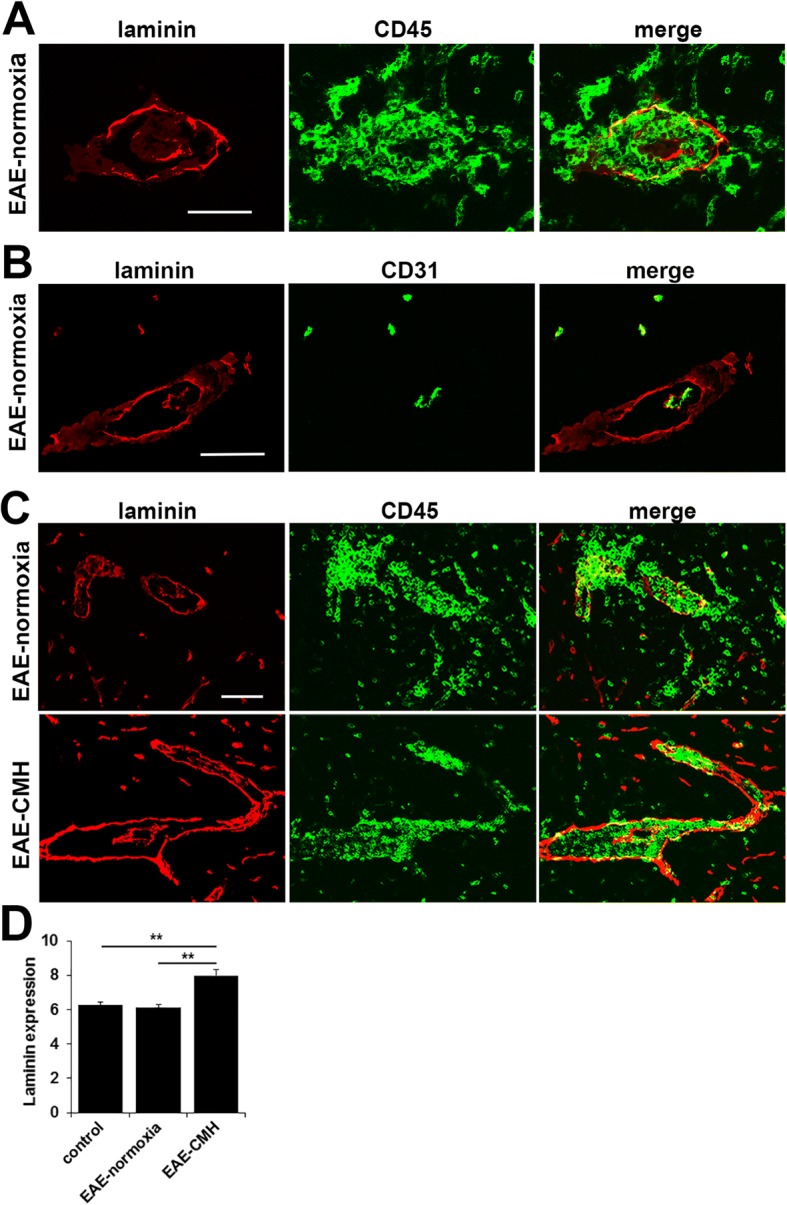
CMH promotes increased laminin expression in the vascular basement membrane. **a**-**c**. Frozen sections of lumbar spinal cord taken from EAE-normoxia or EAE-CMH mice at the peak symptomatic phase of EAE were stained for CD45 (AlexaFluor-488) and laminin (Cy-3) in panel A, CD31 (AlexaFluor-488) and laminin (Cy-3) in panel B or CD45 (AlexaFluor-488) and laminin (Cy-3) in panel C. Scale bar = 50 μm. Note that in EAE, infiltrating leukocytes accumulate in the perivascular space between the endothelial and parenchymal layers of the vascular basement membrane, causing them to separate (**a**). In B note that only the inner layer of basement membrane co-localizes with CD31. In C note that while in the normoxic EAE spinal cord, leukocytes break through the basement membrane to migrate freely into the CNS parenchyma, CMH-treated mice showed a thicker stronger expression of laminin in the basement membrane, resulting in greater containment of leukocytes within perivascular cuffs. **d**. Quantification of vascular laminin expression. Results are expressed as the mean ± SEM (*n* = 6 mice/group). Note that CMH enhanced laminin expression within the parenchymal basement membrane. ** *p* < 0.01

The basement membranes of CNS blood vessels comprise several ECM proteins including laminins, type IV collagen, and fibronectin. In the CNS, vascular basement membranes have two layers, an inner layer contributed by endothelial cells, and an outer parenchymal layer which is contributed by astrocytes and leptomeningeal cells [41]. In the normal CNS, the inner and outer membranes are indistinguishable by light microscopy and appear as one membrane, but during EAE progression, CD45+ leukocytes accumulate in the perivascular space between the two membranes and push them apart, making them clearly distinguishable when stained with a pan-laminin antibody (which recognizes all laminin isoforms; Fig. 5a). Dual-IF with the endothelial marker CD31 and a pan-laminin antibody shows the inner CD31-positive endothelial layer co-staining with laminin, but also highlights the presence of an additional outer laminin-positive layer only when leukocyte perivascular cuffing is present (Fig. 5b).

The laminins are a family of closely related heterotrimeric proteins composed of α, β and γ chains, of which there are 5 α, 3 β and 3 γ chains currently reported, that can combine to form up to 12 different isoforms of laminin [12, 13, 41]. An elegant study performed by the Sorokin lab showed that the two different basement membranes within CNS blood vessels contain different laminin isoforms, with endothelial basement membranes containing laminin-411 (α4β1γ1) and 511 (α5β1γ1), previously known as laminins 8 and 10 respectively, while the parenchymal basement membranes contain laminins-111 (α1β1γ1) and 211 (α2β1γ1), previously known as laminins 1 and 2 respectively [41]. As our initial staining indicated that CD45+ leukocytes appeared to be restrained within perivascular cuffs to a greater extent in CMH-treated mice, we performed dual-IF with CD45 and the pan-laminin antibody to examine this in greater detail. This revealed that while leukocytes in normoxic mice appeared to be initially held back within the perivascular space, over time, leukocytes cross through the parenchymal basement membrane and enter the CNS parenchyma to migrate widely throughout the tissue (Fig. 5c). In contrast, at the same time-point, CMH-treated mice showed much greater leukocyte containment within perivascular cuffs, resulting in far less dispersal into the CNS parenchyma. In addition, while the parenchymal basement membrane in normoxic mice appeared stretched thin and discontinuous in places, in CMH-treated mice it was thick and continuous and showed a much higher level of laminin expression (Fig. 5c). Quantification revealed that while vascular expression of laminin was not changed at the peak stage of EAE under normoxic conditions relative to disease-free controls, levels in EAE-CMH mice at the same time-point were significantly higher compared to disease-free conditions (7.98 ± 0.35 fluorescent units/FOV vs. 6.28 ± 0.17, *p* < 0.01) or EAE-normoxic conditions (7.98 ± 0.35 fluorescent units/FOV vs. 6.10 ± 0.19, *p* < 0.01) (Fig. 5d).

**Fig. 6:**
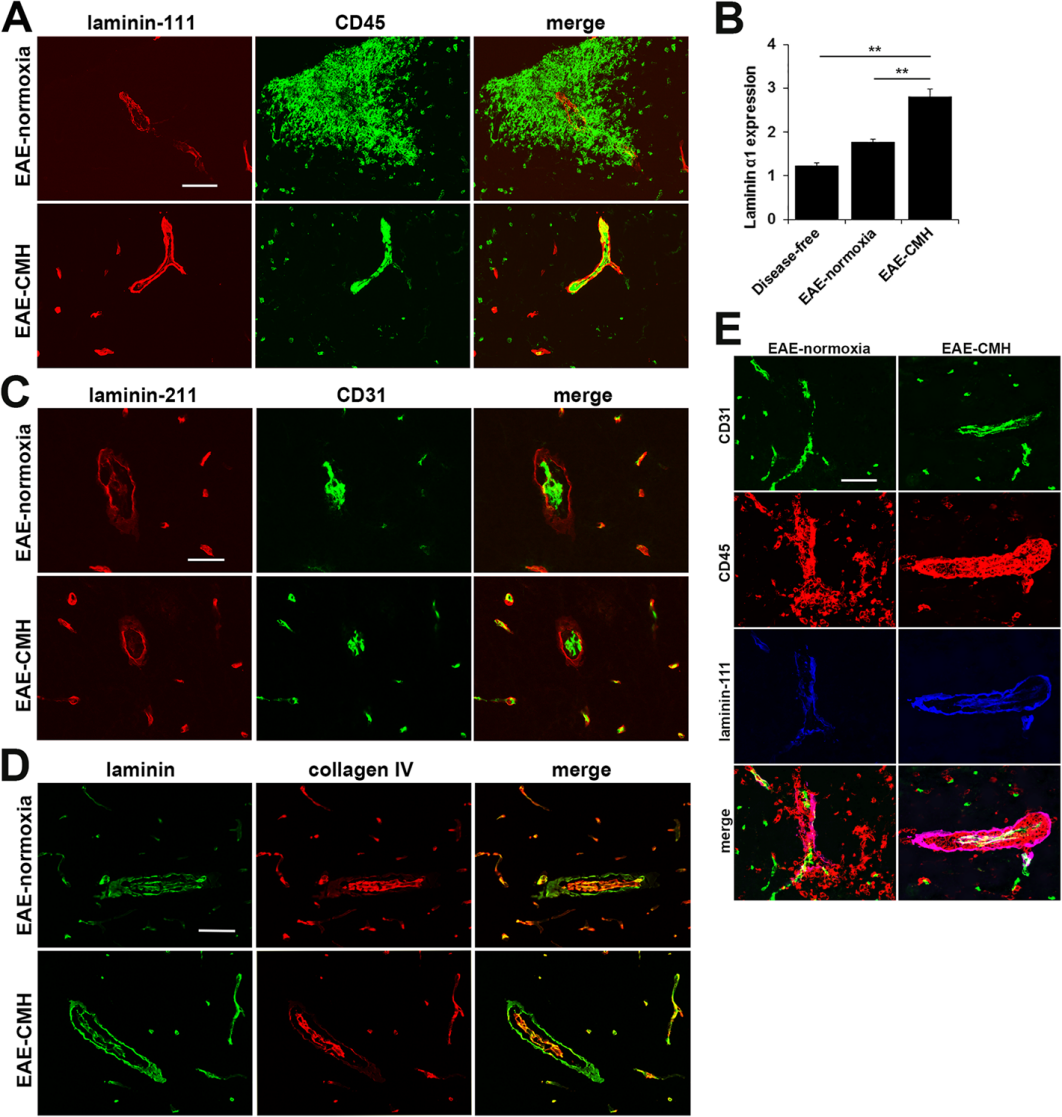
CMH specifically upregulates the laminin isoform-111 in the parenchymal layer of the vascular basement membrane. Frozen sections of lumbar spinal cord taken from EAE-normoxia or EAE-CMH mice at the peak symptomatic phase of EAE were stained for CD45 (AlexaFluor-488) and the laminin α1 subunit (Cy-3) in panel A, CD31 (AlexaFluor-488) and the laminin α2 subunit (Cy-3) in panel C, or type IV collagen (Cy-3) and laminin (AlexaFluor-488) in panel D. Scale bar = 100 μm. **b**. Quantification of vascular laminin α1 subunit expression. Results are expressed as the mean ± SEM (*n* = 6 mice/group). Note that CMH enhanced laminin α1 expression within the parenchymal basement membrane but had no observable effect on laminin α2 expression. Also note that collagen IV is expressed only within the endothelial layer of the vascular basement membrane and expression is not altered by CMH (**d**). ** *p* < 0.01. **e**. CD31/CD45/laminin α1 triple-IF staining of EAE spinal cord under normoxic or CMH conditions. Note that compared to normoxic conditions, the parenchymal vascular basement membrane in CMH-treated mice contains higher levels of laminin-111 and is more effective at restricting the transmigration of CD45+ leukocytes

As parenchymal vascular basement membranes contain the specific laminins-111 and 211 [41], we next performed dual-IF with antibodies against the pan-leukocyte marker CD45 and antibodies specific for the laminin α1 chain or α2 chain to determine which specific laminin isoform accounts for the CMH-induced upregulation of laminin detected with the pan-laminin antibody. As shown in Fig. 6, this revealed that CMH induced strong upregulation of the laminin α1 chain but not α2, corresponding to elevated levels of laminin-111 in the parenchymal basement membrane. Quantification revealed that vascular expression of the laminin α1 chain in EAE-CMH mice was significantly higher than disease-free conditions (2.81 ± 0.16 vs. 1.24 ± 0.05, *p* < 0.01) and EAE-normoxic conditions (2.81 ± 0.16 vs. 1.77 ± 0.07, *p* < 0.01) (Fig. 6b). By contrast, vascular expression of the laminin α2 chain was not appreciably different between EAE-normoxic and EAE-CMH conditions. When we examined the expression of collagen IV, another ECM protein abundantly expressed in vascular basement membranes, we found that expression was predominantly localized to the endothelial layer of the vascular basement membrane (Fig. 6d) and furthermore that CMH did not appreciably alter collagen IV expression levels in the vascular basement membrane. Triple-IF (CD31/CD45/laminin-111) of EAE spinal cord revealed that compared to EAE under normoxic conditions, the parenchymal vascular basement membrane in CMH-treated mice contains higher levels of laminin-111, which closely correlates with reduced transmigration of CD45+ leukocytes out of the perivascular space and into the CNS parenchyma (Fig. 6e). Taken with the previous finding that of all laminin isoforms, laminin-111 is the most inhibitory substrate for T cell adhesion [41], our observations suggest that CMH-induced upregulation of the inhibitory protein laminin-111 in the parenchymal basement membrane keeps leukocytes tightly corralled within the perivascular space, thus preventing their transmigration into the CNS parenchyma.

## Discussion

The goals of this study were to examine the protective potential of hypoxic pre-conditioning in a relapsing-remitting EAE model of MS and then determine how chronic mild hypoxia (CMH) influences vascular integrity and the different cellular and molecular components underlying this integrity. We found that mice exposed to 10% O_2_ (CMH) at the same time as EAE induction, were strongly protected against the development of EAE progression, as assessed both at the clinical and histopathological levels. At the mechanistic level, our studies indicate that CMH protection is mediated at least in part, by enhancing several different properties of blood vessels that contribute to vascular integrity, including (i) reduced expression of the endothelial activation molecules VCAM-1 and ICAM-1, (ii) maintained expression of the endothelial tight junction proteins ZO-1 and occludin, and (iii) enhanced expression of the leukocyte inhibitory protein laminin-111 in the parenchymal layer of the vascular basement membrane. Taken together, these data suggest that hypoxic pre-conditioning protects against EAE by enhancing the integrity of CNS blood vessels at multiple levels.

### The impact of CMH on EAE progression

The data presented here extend previous observations that chronic exposure to mild hypoxia delays the progression of EAE [8, 15]. Our study differs from those reports in that we performed our analysis in the relapsing-remitting form of EAE, in which mice reach peak disease before making significant recovery, and then follow a relapsing-remitting course [32, 33], which more closely resembles the most common form of MS in human patients. With the different models in mind, it is important to note that in the relapsing-remitting model used in our studies, CMH did not just delay EAE progression in the short term, it strongly reduced clinical score both at the peak of disease activity and at all time-points examined thereafter for the duration of the experiment (7 weeks), resulting in a marked and significant reduction in long-term clinical score. This neuroprotective effect of hypoxic pre-conditioning is consistent with a growing number of studies in animal models of ischemic stroke, whereby exposure to mild hypoxia dramatically reduces the size of ischemic infarct [10, 34, 43]. Interestingly, recent studies have highlighted the therapeutic potential of intermittent hypoxic training (IHT) in a number of other experimental neuropathologies, including Alzheimer’s disease, spinal cord injury, epilepsy and ethanol withdrawal-induced stress, raising the notion of therapeutic potential for IHT [17, 19, 24, 31, 48]. While our studies demonstrate protection if hypoxic treatment is started before EAE disease occurs, to our knowledge, no-one has yet examined the impact of CMH on established EAE. In fact, considering that oxygen therapy has been shown to ameliorate clinical disease in rats with established EAE, this suggests that hypoxia applied at this time-point might actually worsen clinical disease [6]. To answer this important question, in future experiments, we plan to investigate the impact of CMH applied at different time-points leading up to peak disease, as well as at peak disease itself and during the remission phase of disease, to more completely define how the timing of application of hypoxic conditioning protects against disease relapse. Regardless of the outcome of our planned studies, it is important to recognize that our demonstration of a strong protective effect of hypoxic pre-conditioning has clear translational significance because it also underlines the importance of using CMH as a biological tool to identify the molecular mechanisms underlying this protection. Using this approach, we have determined that CMH enhances the integrity of CNS blood vessels at multiple levels, including reduced expression of the endothelial activation molecules VCAM-1 and ICAM-1, maintained expression of the endothelial tight junction proteins ZO-1 and occludin, and increased expression of the leukocyte inhibitory ECM protein laminin-111 in the vascular basement membrane.

Recent work suggested that CMH protects against EAE, at least in part, by exerting an immuno-modulatory effect, such that the spinal cords of CMH-treated mice contained reduced levels of Th17+ T cells but increased levels of regulatory T cells (Tregs), thereby promoting an anti-inflammatory milleu in the spinal cord [15]. Our findings extend this data by demonstrating that CMH likely promotes an anti-inflammatory state within CNS blood vessels via a two-pronged attack, first, by exerting an immuno-suppressive effect in the spinal cord, and second by reinforcing the vascular barrier that keeps the immune cells from entering the CNS. While it is possible that the vasculo-protective influence of CMH could be secondary to the immuno-suppressive effect, this seems highly unlikely for the simple reason that even in the absence of immune cell activation (i.e.; no EAE), CMH induces the same marked changes in vascular barrier properties as we observed in our EAE studies, namely enhanced endothelial expression of tight junction proteins and increased laminin-111 expression in the vascular basement membrane [3, 20, 21, 30]. Conversely, turning the question on its head, is it possible that the creation of an immuno-suppressed state in the spinal cord occurs secondary to changes in vascular integrity or endothelial activation state? Recent studies indicate that CMH does not directly influence immune cell activation in EAE as T cell sensitization to the myelin antigen is not altered but rather the number of Th17+ T cells that infiltrates the spinal cord is reduced [15]. This suggests that the most important determinant of the inflammatory state within the spinal cord is the manner in which blood vessels regulate the transit of the different T cell populations across the BBB. Our finding that CMH suppresses endothelial expression of the key activation molecules VCAM-1 and ICAM-1 strongly suggests that CMH reduces endothelial activation state, which together with enhanced barrier integrity due to increased expression of tight junction proteins and laminin-111 in the basement membrane, acts to reduce the influx of pro-inflammatory Th17+ T cells. Taken together, this implies that the pivotal mechanism underlying CMH protection from EAE resides at the level of enhanced BBB integrity and reduced endothelial activation state.

### The influence of CMH on vascular expression of tight junction proteins during EAE

Tight junction proteins are a major determinant of the BBB [1, 23, 38]. As previous studies have shown that expression of some tight junction proteins is lost in MS and EAE lesions [2, 14, 25], but upregulated in response to CMH [3, 20, 30], this suggested that CMH might be conferring protection via influencing tight junction protein expression. Significantly, while our studies revealed that both ZO-1 and occludin are universally downregulated on all spinal cord blood vessels at the peak stage of EAE, mice treated with CMH were protected against loss of these two tight junction proteins. Interestingly, a recent study suggested a direct connection between vascular laminin and tight protein expression by showing that deletion of astrocyte laminin resulted in decreased expression of tight junction proteins at the BBB [47]. This implies that regulation of the three different components of the BBB we have described here may not be working independently of each other, but more likely are coordinated in a synchronous manner to achieve the same goal.

### The influence of CMH on laminin expression in vascular basement membranes during EAE

Our data show that CMH stimulates upregulation of laminin-111 in the parenchymal basement membrane, and that this correlates with containment of infiltrating leukocytes within the perivascular space, resulting in less migration into the CNS parenchyma. The Sorokin lab has performed extensive studies to examine the roles of basement membrane laminins in restricting leukocyte infiltration across blood vessels during EAE pathogenesis. In particular, they focused on the laminins-411 and 511 that are expressed specifically within the endothelial layer of the vascular basement membrane, and found that while leukocytes migrate freely across laminin-411, they migrate less well across points in the vessel where laminin-511 is expressed, suggesting that laminin-511 may inhibit leukocyte migration [41]. This idea was later reinforced by the finding that mice deficient in laminin-411, which show laminin-511 expression distributed throughout all vessels, show reduced levels of EAE and leukocyte infiltration [46]. Our findings extend these studies by showing that not only are endothelial laminins important in regulating the passage of infiltrating leukocytes, but that laminins present in the parenchymal basement membrane, particularly laminin-111, may also play an important role. Indeed, as leukocytes appear to cross the endothelial layer relatively easily during EAE, but then get held up by the parenchymal basement membrane in the perivascular space as a result of hypoxic pre-conditioning, our findings suggest that the parenchymal basement membrane may actually be the critical gatekeeper regulating leukocyte entry into the CNS. Consistent with this idea, of all the different laminins, in vitro studies have shown that laminin-111 is the least permissive for leukocyte adhesion [41], suggesting that this specific laminin represents a robust barrier to leukocyte transmigration. Indeed, it is noteworthy that laminins-111 and 211 are only expressed in vascular basement membranes in CNS blood vessels and not detected in other vascular beds, implying that the CNS may have evolved this unique mechanism to enhance vascular integrity as a way of limiting leukocyte infiltration into the CNS.

In conclusion, in this study we have shown that CMH strongly protects against the development of EAE progression, as assessed both at the clinical and histopathological levels. Our mechanistic studies reveal that CMH protection tightly correlates with enhancement of several different properties of blood vessels that contribute to vascular integrity, including reduced endothelial expression of the activation molecules VCAM-1 and ICAM-1, enhanced endothelial expression of the tight junction proteins ZO-1 and occludin, and increased expression of the leukocyte inhibitory protein laminin-111 in the parenchymal layer of the vascular basement membrane. Together, these data suggest that hypoxic pre-conditioning protects against EAE by enhancing the integrity of CNS blood vessels at multiple different levels.

## Abbreviations

BBB: Blood-brain barrier
BSCB: Blood-spinal cord barrier
CNS: Central nervous system
Dual-IF: Dual-immunofluorescence
EAE: Experimental autoimmune encephalomyelitis
ECM: Extracellular matrix
FOV: Field of view
MS: Multiple sclerosis
PLP: Proteolipid protein
SEM: Standard error of the mean
ZO-1: Zonula occludens-1
